# Mechanically Robust Dual-Crosslinking Elastomer Enabled by a Facile Self-Crosslinking Approach

**DOI:** 10.3390/ma15113983

**Published:** 2022-06-03

**Authors:** Zhendong Huang, Biqiang Jin, Haitao Wu, Zihang Zeng, Minghui Huang, Jinrong Wu, Lusheng Liao, Jing Zheng

**Affiliations:** 1 State Key Laboratory of Polymer Materials Engineering, College of Polymer Science and Engineering, Sichuan University, Chengdu 610065, China; zhendonghuang@yeah.net (Z.H.); elden_jin@163.com (B.J.); wht2281007108@163.com (H.W.); zeng.zihang@foxmail.com (Z.Z.); 18156942758@163.com (M.H.); wujinrong@scu.edu.cn (J.W.); 2 Guangdong Provincial Key Laboratory of Nature Rubber Processing, Agricultural Products Processing Research Institute of Chinese Academy of Tropical Agricultural Science, Zhanjiang 524001, China

**Keywords:** elastomers, dual-crosslinking, hydrogen bonds

## Abstract

We propose a simple but rapid strategy to fabricate self-crosslinked dual-crosslinking elastomers (SCDCEs) with high mechanical properties. The SCDCEs are synthesized through one-pot copolymerization of Butyl acrylate (BA), acrylic amide (AM), and 3-Methacryloxypropyltrimethoxysilane (MEMO). Both the amino group on AM and the methoxy group on MEMO can be self-crosslinked after polymerization to form a dual-network crosslink consisting of hydrogen bonds crosslink and Si-O-Si covalent bonds crosslink. The SCDC endow optimal elastomer with high mechanical properties (the tensile strength is 6MPa and elongation at break is 490%) as the hydrogen bonds crosslink can serve as sacrificial construction to dissipate stress energy, while covalent crosslinking networks can ensure the elasticity and strength of the material. These two networks also contribute to the recoverability of the elastomers, leading them to recover their original shape and mechanical properties after being subjected to deformation in a short time.

## 1. Introduction

Elastomers are widely used in various fields such as tires, biomaterials, seals, wearable electronic devices, soft robots, sensors, and other fields [1,2,3,4]. These require elastomers with high mechanical properties. Hence, it is important to seek for ways to fabricate and toughen elastomers. There are many toughening strategies of elastomers: (i) incorporating nanofillers to introduce high-level energy dissipation through friction and the break of filler network; (ii) constructing special structures such as hard and soft segments in polyurethane to form microphase separation; (iii) introducing sacrificial bonds, which can improve the toughness of elastomers by dissipating energy and redistributing stress before the failure of elastomers [5,6,7,8]. Among them, introducing sacrificial bonds is an emerging and effective strategy [9,10,11].

To introduce sacrificial bonds, there are methods including constructing double networks, dual crosslinking, and sacrificial skeleton [12,13,14]. Inspired by the work of double networks hydrogels, Luo et al. introduced 2-ureido-4[1H]-pyrimidinone (UPy) motifs which dimerize and form strong hydrogen bonds, creating covalently crosslinked polyisoprene (PI) elastomers. Elastomers toughened by UPy groups show higher toughness and mechanical strength than those toughened by hydroxyl groups [15]. Peng et al. synthesized a strong, tough dual crosslinking elastomer consisting of ionic bonds and Diels–Alder (D-A) bonds. The ionic bonds serve as sacrificial bonds through electrostatic interaction, while the D-A crosslinks confer elasticity [16]. Zhu et al. designed a sacrificial skeleton, combing a microscopic three-dimensional inverse opal-mimetic skeleton of glutaraldehyde cross-linked chitosan and a continuous matrix of sulfur-vulcanized natural rubber, and fabricated defect-tolerance, strong, and tough soft materials [17]. Nevertheless, all the strategies, including double networks, dual crosslinking, and sacrificial skeleton, hold a critical drawback which is the complexity of synthesis. Therefore, it is necessary to find a way to reach a rapid synthesis of elastomers with high mechanical properties, including sacrificial structures.

To simplify the preparation steps of polymers, self-crosslinking is usually a good approach. This approach has been used to fabricate coating, adhesives, hydrogels, etc. [18,19,20,21]. However, this self-crosslinking has not been explored to fabricate dual-crosslinking elastomers. For example, adding silane coupling agents is a popular self-crosslinking method which is usually used to prepare an acrylic emulsion [18]. Therefore, we can envision that the organofunctional siloxane introduced in the polymer backbone chain may self-crosslink and form covalent bond networks. If other monomers which provide sacrificial bonds are also polymerized in the backbone chain, a dual networks elastomer with high mechanical properties may be conveniently fabricated.

In this paper, we developed a self-crosslinking approach toward the facile construction of dual-crosslinking elastomers with high mechanical properties. The silane coupling agent and acrylamide (AM) were introduced into the polymer chain, making the polymer able to self-crosslink under an ambient environment. Thus, the crosslinked elastomer with hydrogen bonds formed by amino and Si-O-Si covalent bonds, which are self-crosslinked, can be simply prepared just after polymerization. In our material, hydrogen bonds act as sacrificial bonds to toughen the elastomers and enable the elastomers to be self-healable; the self-crosslinking Si-O-Si bonds maintain the shape of the elastomers and provide strength. In addition, this dual-crosslinked network possesses ideal dynamic nature, leading to the rapid self-recovery of elastomers. In general, the elastomers show high mechanical properties (the tensile strength is 6MPa and elongation at break is 490%) and fast self-recoverability.

## 2. Materials and Methods

### 2.1. Materials

2, 2-azobisisobutyronitrile (AIBN) was provided by Kermel (Tianjin, China). Butyl acrylate (BA, stabilized with Hydroquinone monomethylether, >99.0%, TCI, Shanghai, China), acrylic amide (AM, >98%, Alfa Aesar, Shanghai, China), 3-Methacryloxypropyltrimethoxysilane (MEMO, >98%, Adamas, Shanghai, China), Tetrahydrofuran (THF), 1,4-dioxane, n-hexane, and petroleum ether were purchased from Tansoole.com (accessed on 22 July 2021) (Shanghai, China). BA was filtrated through a basic alumina column to remove the inhibitor, while other reactants were used without further purification.

### 2.2. Synthesis of the SCDCEs

The facilely constructed self-crosslinking dual crosslinking elastomers (SCDCEs) were synthesized simply by free-radical copolymerization of BA, AM, and MEMO. The details of polymerization were as follows: firstly, 8.88 g (0.0692 mol) BA, 2.13 g (0.03 mol) AM, 0.19 g (0.0008 mol) MEMO, and a bit of AIBN were dissolved in 30 mL THF. Additionally, then it is transferred to a 150 mL there-neck round bottom flask equipped with an economical allihn condenser and a magnetic stirrer. Dissolved oxygen in the mixture was removed by bubbling argon for at least 20 min. The monomers were then polymerized under an argon atmosphere with stirring at 70 °C for 6 h. After polymerization, the product was precipitated in petroleum ether or n-hexane at least three times. The precipitate was then dissolved in the 20 mL 1,4-dioxane and then transferred to a square Teflon mold in the air. After the polymer networks were self-crosslinked at 25 °C in air for 24 h, the samples were put into a vacuum oven at 60 °C for 7 days to evaporate the solvent. After this process, transparent films were obtained (Figure 3a).

### 2.3. Characterization

The FTIR spectra were measured by an attenuated total reflection mode at room temperature using a Thermo Scientific (Waltham, MA, USA) Niolet iS50 FTIR. Thermogravimetric analysis (TGA) was performed on a NETZSCH TG209F1 thermogravimetric analyzer. The heating rate was 10 °C min^−1^. A TA Instruments Q1000 was used to perform the differential scanning calorimetry (DSC) measurements in the temperature range of −70 °C to 60 °C (10 °C min^−1^). Raman spectra (DXR, Thermo Fisher Scientific, Waltham, MA, USA) were performed to confirm the characteristic peaks with a DXR laser (532 nm). A SPI4000 AFM instrument of Seiko using silicon tips (NSG10) was used to perform atomic force microscopy (AFM) tests. The spring constant of 3 N m^−1^ was at a resonance frequency of 228.9 kHz. Additionally, the measurements were all carried out in tapping (AC) mode. The rheological measurements were carried out using a TA AR2000ex rotational rheometer (TA instrument Ltd., New Castle, DE, USA). The samples were 8 mm in diameter and 0.9–1.1 mm thick and were cut into disks. Stress relaxation tests were performed at a strain of 0.1% for 40 s. Temperature sweep tests were performed on the samples at a wide temperature range of 25 °C to 180 °C with a strain of 0.1%. Strain scanning tests were performed at a constant frequency of 1.0 Hz. Periodic structural failure and reconstruction tests were performed on SCDCEs with periodic strain (0.1% and 50%) at a constant frequency of 1.0 Hz. A universal testing machine (Instron 5967) was used to measure mechanical properties. To obtain the stress–strain curves and cyclic tension curves, all the tests were carried out with a strain rate of 0.083 s^−1^ at room temperature.

## 3. Results and Discussion

### 3.1. Fabrication and Structure

**Figure 1:**
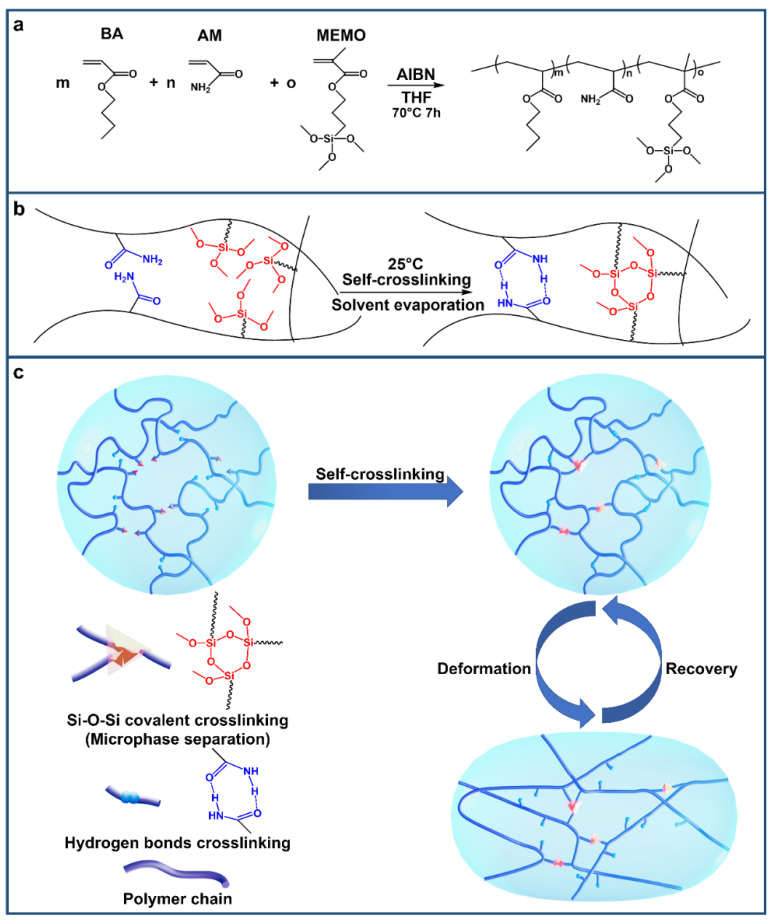
Design concept and synthesis. (**a**) Synthesis of the SCDCE: copolymerize BA, AM, and MEMO by one-pot free-radical copolymerization with AIBN initiator. (**b**) Self-crosslink. (**c**) Schematic description of self-crosslinking mechanisms and proposed mechanisms for the energy dissipation of SCDCs under deformation.

**Figure 2:**
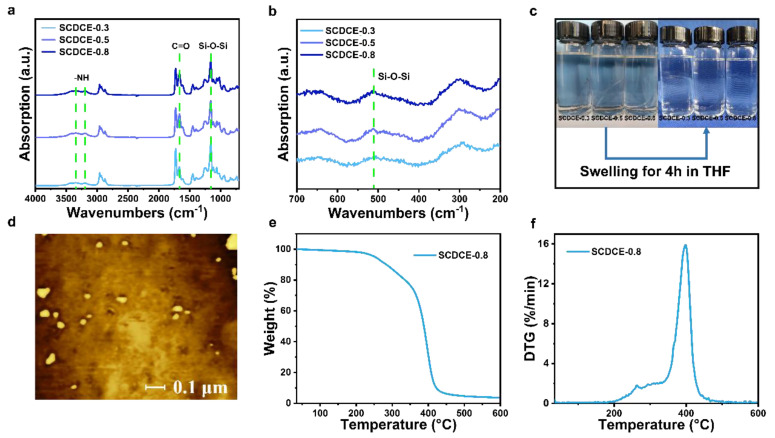
Structure analyzing and thermal properties. (**a**) FTIR spectrum of the SCDCEs samples. (**b**) Raman spectrum of SCDCEs. (**c**) Photograph of SCDCEs swelling in THF. (**d**) AFM phase image of SCDCE-0.8. TGA (**e**) and DTG (**f**) curves of SCDCE-0.8.

**Figure 3:**
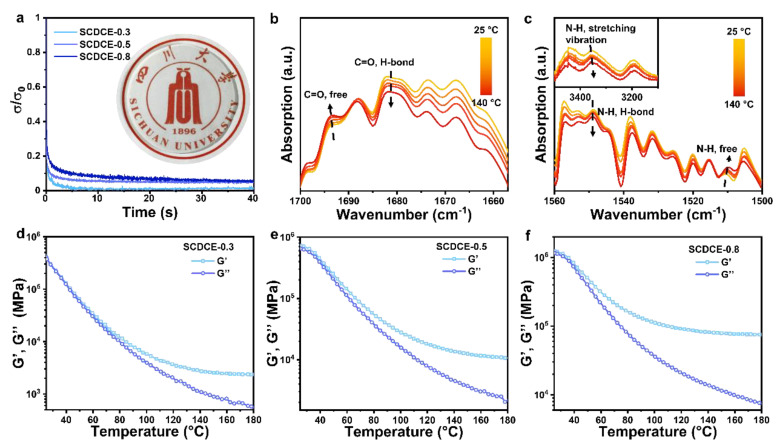
Dynamic properties. (**a**) Stress relaxation curves of SCDCEs samples. The samples are stretched to a strain of 0.1% for 40 s. Temperature-dependent FTIR spectra at (**b**) 1700–1660 cm^−1^, (**c**) 1580–1500 cm^−1^ and 3500–3100 cm^−1^ of SCDCE-0.3 upon heating from 25 °C to 140 °C. (**d**–**f**) Dependence of the SCDCEs samples G′ and G″ moduli on temperature. The samples are stretched to a strain of 0.1% at a constant frequency of 1.0 Hz.

The SCDCEs are simply synthesized through the one-pot free-radical copolymerization of butyl acrylate (BA) acrylic amide (AM) and 3-Methacryloxypropyltrimethoxysilane (MEMO) with azobisisobutyronitrile (AIBN) initiator, resulting in an uncrosslinked copolymer (Figure 1a). The amino group of the AM molecule can provide hydrogen bonds as sacrificial bonds. Additionally, the methoxy groups on the MEMO can be self-crosslinked in the air to form a Si-O-Si bond crosslinking network. So SCDCEs can be prepared through only one step. After polymerization, the uncrosslinked polymer only needs to be self-crosslinked in the air for 24 h to obtain a covalent network crosslinked by Si-O-Si bonds (Figure 1b). Moreover, the amine groups of AM are evenly distributed on the polymer backbones, forming hydrogen bonds with each other and dispersively aggregating. These form hydrogen bond crosslinkings, which are dynamic and reversible (Figure 1b,c). Based on the above design mechanism, a series of samples were fabricated by changing the amount of MEMO to control the ratio of the two crosslinks. The self-crosslinked double-crosslinking elastomers are denoted as SCDCE-x, where x represents the percentage of MEMO (mol, %). The random copolymerization makes the two crosslinks disperse from each other and results in a transparent elastomer (Figure 2c and Figure 3a). It can be envisioned that the dynamic hydrogen bond crosslinking can separate gradually and dissipate a great amount of energy during deformation, whereas covalent Si-O-Si bond crosslinking preserves the elasticity of the elastomer thus allowing them to recover their original shape upon stress relief. The hydrogen bonds can be reformed during the recovery process, making SCDCEs self-healable (Figure 1c).

The structures of the SCDCEs were characterized by Fourier transform infrared spectroscopy (FTIR). It can be found that the SCDCEs samples exhibit several characteristic peaks: the absorption peaks at 3350 cm^−1^ and 3180 cm^−1^ indicate the presence of N-H stretching vibration [22,23,24]; the peak at 1680 cm^−1^ can be attributed to the C=O stretching vibration [23,24]; the absorption peak at 1200 cm^−1^ can be assigned to the Si-O-Si bonds [25]. To verify that the methoxy groups of MEMO are cross-linked to form Si-O-Si bonds, the Raman spectrum of SCDCEs was obtained (Figure 2b). The absorption peak at 540 cm^−1^ belongs to the Si-O-Si bonds, demonstrating that the methoxy group of MEMO reacted to each other and was self-crosslinked by forming Si-O-Si bonds [26]. Moreover, an atomic force microscope (AFM) image (Figure 2d) reveals that Si-O crosslinks could self-assemble nanoparticles, forming microphase separations in SCDCEs (which can improve mechanical properties). This microphase separation may broaden the glass transition region of the SCDCEs [27]. The glass transition temperature (*T_g_*) values of SCDCEs obtained from the differential scanning calorimetry (DSC) curve lie within the range from −19 °C to 3 °C (Figure S1), which confirms that the materials are rubbery rather than glassy plastics at room temperature. The TGA and DTG curves of SCDCE-0.8 are shown in Figure 2e–f. The main weight loss occurred in the temperature range of 350–400 °C. The weight loss of the sample did not reach 5% until 250 °C, indicating that the sample is resistant to high temperatures to a certain extent.

### 3.2. Dynamic Nature of the Dual Crosslinks

The dynamic nature of the dual-network elastomers is investigated by performing stress relaxation experiments. The samples are tested at the strain of 0.1%, and the results are shown in Figure 3a. It is obvious that when the dynamic network relaxes, and the force exerted on this network is released, SCDCE exhibits an apparent relaxation behavior. Furthermore, the more applied force that is released refers to the higher molar ratio of MEMO addition. This is attributed to the presence of Si-O-Si bond crosslinks which are covalent permanent crosslinks.

### 3.3. Mechanical Properties

**Figure 4:**
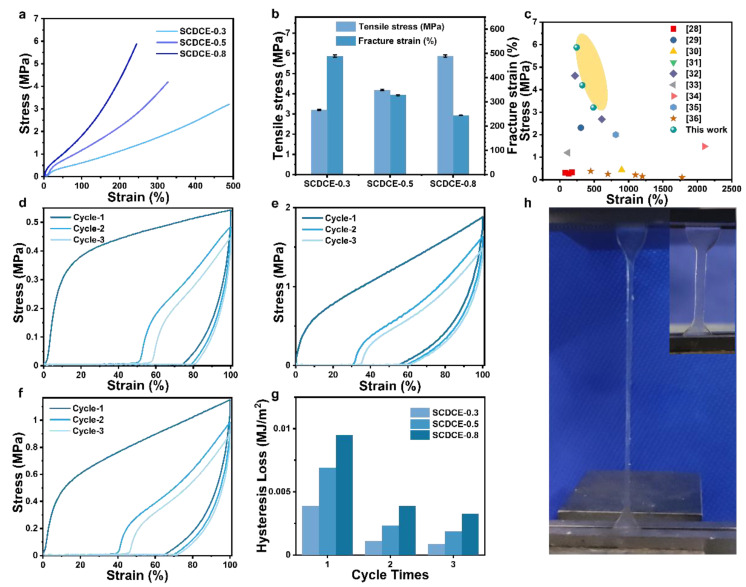
Mechanical properties. (**a**) Tensile tests of SCDCEs samples at a strain rate of 0.083 s^−1^. (**b**) Summary of mechanical properties of the SCDCEs. (**c**) Ashby plot of the mechanical properties of SCDCEs and recently reported acrylate-based elastomers [28,29,30,31,32,33,34,35,36]. Cyclic stress–strain curve of SCDCE-0.3 (**d**), SCDCE-0.5 (**e**) and SCDCE-0.8 (**f**). (**g**) Schematic photo of SCDCEs stretching process.

To characterize the mechanical properties of the SCDCEs, the tensile tests were carried out at a strain rate of 0.083 s^−1^ on dumbbell-shaped samples. Representative stress–strain curves are shown in Figure 4a. It can be noted that as the MEMO content increases, the intensity of SCDCEs increases, but stretchability decreases (Figure 4b). The breaking strength of SCDCE-0.3 is 3 MPa, and the elongation at break is 490%. When the MEMO content increased to 0.6% and 0.8%, the tensile strength of the elastomer increased to 4 MPa and 6 MPa, and the elongation at break decreased to 300% and 200%. It can be inferred that, with the increase of MEMO content, Si-O-Si covalent cross-linking between polymer molecules increases, leading to an increase in strength but a decrease in elasticity.

To further explore the excellent mechanical properties of SCDCEs, cyclic tensile experiments were carried out for SCDCEs (Figure 4d–f). The first-time cyclic stress–strain curves of SCDCEs have large hysteresis loss, which indicates a large amount of energy dissipation. However, during the cyclic loading and unloading process, the stress on reloading is less than that on the initial loading for the same strain. This stress softening phenomenon is referred to as the well-known Mullins effect [37,38]. Obviously, upon deformation, the hydrogen crosslinks should preferentially break before the fracture of Si-O-Si covalent crosslinks. It can be speculated that the reversible break and reformation of hydrogen crosslinks lead to energy dissipation [15], improving the mechanical properties of SCDCEs. The integrated area of the hysteresis loop is calculated to quantify the energy dissipation, as shown in Figure 4g. It can be seen that in the first cycle, the hydrogen bond breaks as a sacrificial construction, resulting in huge hysteresis loss. The hydrogen bond could not be repaired immediately during the second and third cycle stretching, and the area of the hysteretic loop decreased to 20–40% of the original, indicating that the hydrogen bond cross-linking network greatly improved the toughness of SCDCEs. At the same time, with the increase of MEMO addition, the proportion of the Si-O-Si covalent bond cross-linking network was increased, and the strength of SCDCEs was increased. Together, these two networks give high mechanical properties to SCDCEs.

### 3.4. Self-Recoverability

**Figure 5:**
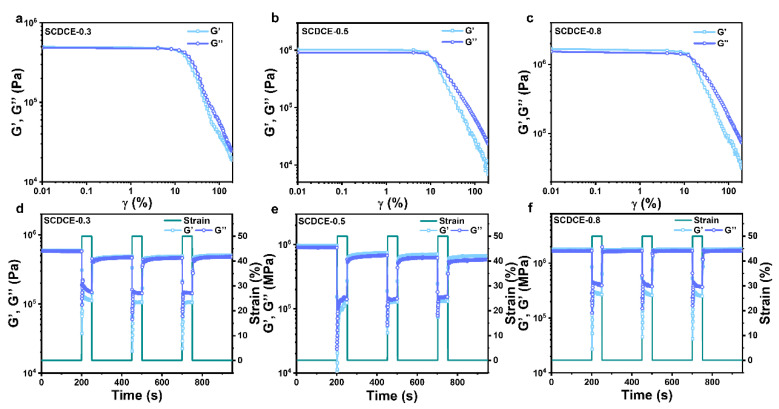
Self-recoverability. (**a**–**c**) G′ and loss G″ moduli vs. strain of SCDCEs at a constant frequency of 1.0 Hz. (**d**–**f**) G′ and G″ moduli vs. periodic strain (0.1% and 50%) of SCDCEs at a constant frequency of 1.0 Hz.

In practical use, it is required that the elastomer can recover its original shape and mechanical properties after being subjected to deformation. To further investigate the rapidly constructed dual-crosslinking structure, we investigated the network restoration and reconstruction tests. Firstly, strain scanning tests were carried out to determine the linear viscoelastic region of SCDCEs, and the results were obtained as shown in Figure 5a–c. G′ and G″ values of the three samples of SCDCEs remain constant in the strain range of 0–10%, indicating that no obvious damage occurred in the three samples at this stage. Moreover, the values of G′ and G″ in the three samples were positively correlated with the proportion of Si-O-Si bonds crosslinking network, and the values of G′ and G″ in SCDCE-0.8 were greater than those in SCDCE-0.5 which were also greater than those in SCDCE-0.3. When the strain exceeded 10%, the values of G′ and G″ of the three samples began to decrease, indicating that the network structure began to be damaged.

After that, the structural recovery and reconstruction performances of SCDCEs were analyzed through periodic structural failure and reconstruction tests. The three samples were tested with an alternate small strain (γ = 0.1%, frequency = 1.0 Hz) and a large one (γ = 50%, frequency = 1.0 Hz). It can be observed from Figure 5d–f that when the strain drops from 50% to 0.1%, the values of G′ and G″ of the three samples nearly return to the original level, and SCDCE-0.8 can immediately restore the values of G′ and G″ that are almost the same as the original. It shows that after the crosslinking network of SCDCEs is destroyed, it can be rebuilt in a very short time to recover to the original performance. More interestingly, after the strain dropped from 50% to 0.1%, the values of G’ and G″ of the three samples increased to a constant state in a short time gradually, indicating that there was a certain time depending on the network restoration and reconstruction. This time dependence is more obvious when the proportion of Si-O-Si bonds crosslinking network is smaller, as more hydrogen bonds crosslinking networks are needed to repair and rebuild the network, which means it takes more time to approach the original performance of the sample. In general, this dual-crosslinking structure can achieve efficient restoration and reconstruction in a short time after being damaged, which is related to the ratio of the two kinds of cross-linked networks.

The small strain recoverability of SCDCEs was obtained from the periodic structural failure and reconstruction tests. To investigate the recoverability of SCDCEs stretched to a large strain, a sample of SCDCE-0.8 was stretched to a strain of 300% (Figure S2). After releasing the stress, the instant residual strain was 150%, and it decreased gradually as a function of time. The sample recovered fast, and it took only 120 s to achieve 90% recovery. Approximately 4600 s were taken to recover to the original shape with no evident residual strain. This rapid recoverability of the sample demonstrates the excellent ability of SCDCEs to retain the original shape of the elastomers after deformation.

## 4. Conclusions

In conclusion, we developed a self-crosslinking approach toward the facile construction of dual-crosslinking elastomers and synthesized a series of elastomers with high mechanical properties. We introduced MEMO and AM onto the polymer backbone, enabling the elastomer to form a self-crosslinking double crosslink. The SCDN consists of Si-O-Si covalent crosslinks and hydrogen bonds crosslinks. During stretching, the hydrogen bonds can serve as sacrificial structures which can dissipate energy and redistribute stress before the failure of elastomers. This leads to the high mechanical properties of SCDCEs. The Si-O-Si covalent crosslinks can maintain the network. We found that the increased proportion of siloxane can increase the mechanical properties of the SCDCEs. As a result, the SCDCs process leads to high strength (6 MPa), high stretchability (490%), and the fast recovery of elastomers.

## Data Availability

The data presented in this study are available on request from the corresponding author.

## Supplementary Materials

The following supporting information can be downloaded at: https://www.mdpi.com/article/10.3390/ma15113983/s1. Figure S1: DSC curves of SCDCEs samples under N_2_ in the temperature range of −70 °C to 60 °C at a heating scan rate of 5 °C min^−1^; Figure S2: Recovery time of SCDNE-0.8 after stretched to the strain of 300%.
