# Supplementary material for: Mechanically Robust Dual-Crosslinking Elastomer Enabled by a Facile Self-Crosslinking Approach
Source: Materials (Basel). 2022 Jun 3;15(11):3983. doi: 10.3390/ma15113983 (PMC9182282; doi:10.3390/ma15113983)
Supplement: Supplementary file 1 [file materials-15-03983-s001.zip › materials-1748296-supplementary.pdf]

Mechanically robust dual-crosslinking elastomer enabled by a facile self-crosslinking approach

Zhendong Huang[a], Biqiang Jin[a], Haitao Wu[a], Zihang Zeng[a], Minghui Huang[a], Jinrong Wu[a], Lusheng Liao[b*], and Jing Zheng[a*]

[a] State Key Laboratory of Polymer Materials Engineering, College of Polymer Science and Engineering, Sichuan University, Chengdu 610065, China

[b] Guangdong Provincial Key Laboratory of Nature Rubber Processing, Agricultural Products Processing Research Institute of Chinese Academy of Tropical Agricultural Science, Zhanjiang 524001, China

Corresponding author contact:

Associate Professor Jing Zheng

State Key Laboratory of Polymer Materials Engineering, College of Polymer Science and Engineering, Sichuan University, Chengdu 610065, China

Email: zhengjing@scu.edu.cn

Professor Lusheng Liao

Guangdong Provincial Key Laboratory of Nature Rubber Processing, Agricultural Products Processing Research Institute of Chinese Academy of Tropical Agricultural Science, Zhanjiang 524001, China

Email: 276491692@qq.com

Supplementary data

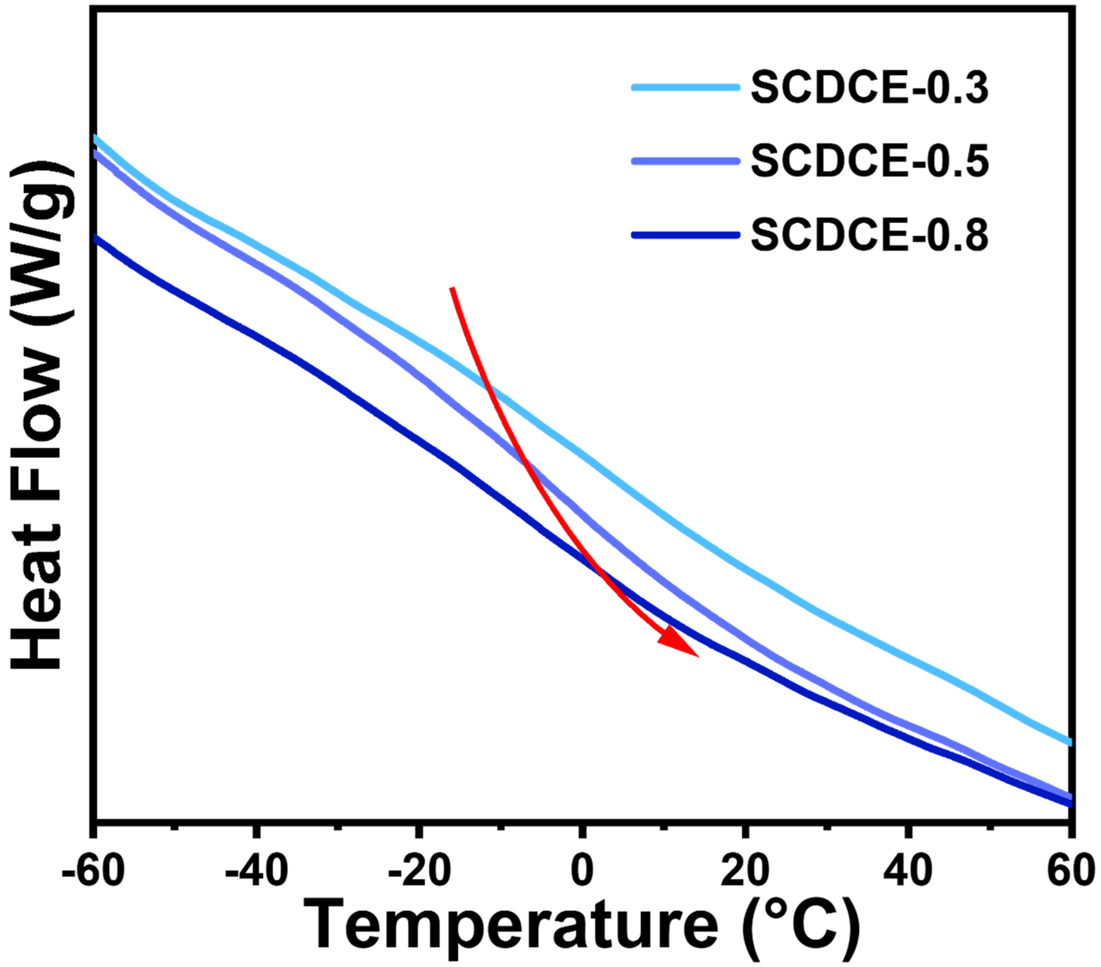

Figure S1. DSC curves of SCDCEs samples under N_2 in the temperature range of -70 °C to 60 °C at a heating scan rate of 5 °C min^{-1}.

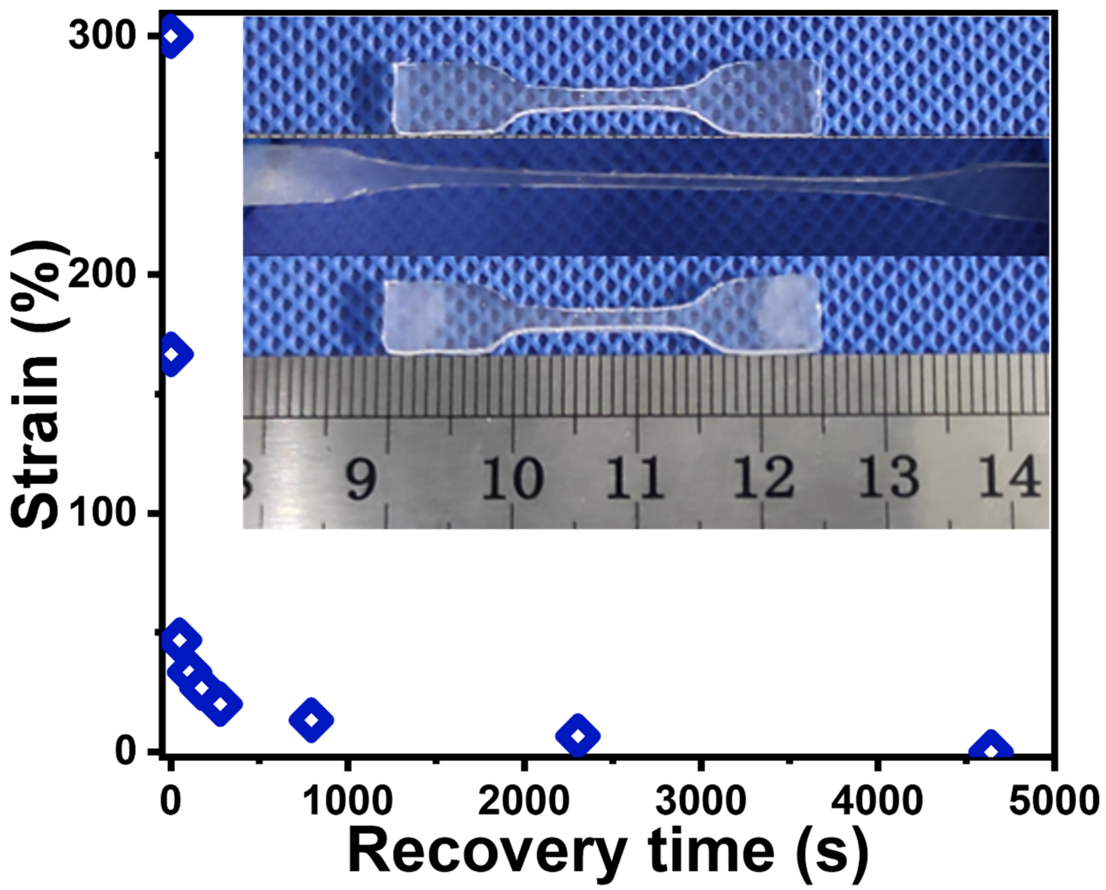

Figure S2. Recovery time of SCDNE-0.8 after stretched to the strain of 300%.
